# Evaluation of Triple Neurotization Technique as a Single Procedure in Adult Traumatic Brachial Plexus Injury

**DOI:** 10.7759/cureus.6660

**Published:** 2020-01-15

**Authors:** Shady Hermena, Tarek El-Gammal, Amr El-Sayed, Mohamed M Kotb

**Affiliations:** 1 Trauma and Orthopedics, Yeovil District Hospital, Yeovil, GBR; 2 Trauma and Orthopedics, Assiut University Hospital, Assiut, EGY

**Keywords:** brachial plexus injury, triple neurotization

## Abstract

Introduction: Brachial plexus injuries are common and result in significant disabilities. This study evaluated the outcome of triple neurotization as a single procedure for upper trunk brachial plexus injury.

Patients and Methods: Some 25 adult consecutive patients with injured upper trunk brachial plexus who underwent microscopic reconstructive surgery using triple neurotization technique in the authors’ institute were recruited in this study. Data on operative and functional outcomes were captured. Modified Narkas scale was used to evaluate the shoulder function in addition to Waikakul scale which was used to evaluate the elbow function. Data were analyzed with respect to short and long term with a median follow-up duration of two years.

Results: Assessment of the recovered shoulder abduction was excellent in 48% (n=12), good in 24% (n=6), fair in 16% (n=4), and poor in 12% of cases (n=3). Shoulder external rotation recovery was excellent in 48% (n=12), good in 12% (n=3), fair in 12% (n=3), and poor in 28% of cases (n=7). Recovery of elbow flexion was excellent in 60% (n=15), good in 12% (n=3), fair in 12% (n=3), and poor in 16% of cases (n=4). The mean value of recovered shoulder abduction was 111.26 degrees (range: 70-150). The mean value of restored shoulder external rotation was 57.5 degrees (range: 45-70). The mean value of restored elbow flexion was 75 degrees (range: 55-120).

Conclusion: Triple neurotization technique can be effective to restore elbow flexion, shoulder abduction, and external rotation in adult patients with upper trunk brachial plexus injury.

## Introduction

Adult traumatic brachial plexus injuries are common injuries resulting in significant disabilities [1]. Most of these injuries are caused by closed traction or compression forces, causing nerves avulsion, rupture, or stretching [2]. However, it can also be caused by open mechanisms, either iatrogenic injury during mastectomy or rib resection surgeries or stab injuries. The optimal timing for reconstructive brachial plexus procedures is within six months [3]. Surgery delayed for more than 18-24 months usually results in poor outcomes due to muscle fibers fatty degeneration [4]. Conservative treatment outcomes are usually unsatisfactory [5-10]. Triple neurotization means nerve transfer of spinal accessory nerve to suprascapular nerve, ulnar fascicles to biceps branch of musculocutaneous nerve, and triceps long or lateral head branch to axillary nerve to restore elbow ﬂexion, shoulder abduction, and external rotation. There is little evidence on this technique in adult traumatic brachial plexus injuries. The purpose of this study was to evaluate the results of triple neurotization as a single procedure to restore shoulder abduction, external rotation, and elbow flexion after upper trunk brachial plexus injury.

## Materials and methods

After ethical approval by our institution's Human Studies Committee, a retrospective chart review was performed. Adult post-traumatic brachial plexus palsy patients were included in this study with no secondary reconstruction around the shoulder before or after the nerve transfer. Patients were followed up for at least 18 months.

Operative technique

The standardized operative protocol was followed, which involved positioning patients supine with prepared and draped both legs in case sural nerve graft was needed. During the first stage, an incision was made along the posterior edge of the sternocleidomastoid muscle and parallel to the lower border of the clavicle to expose the supraclavicular part of brachial plexus. Then a 10-cm incision was made on the anteromedial aspect of the arm, and the musculocutaneous nerve was followed to find the biceps motor branch which was dissected free and divided for about 2 cm from entry to the muscle. The ulnar nerve was also identified through the same incision and dissected using microscopic magnification to isolate a motor fascicle. The chosen fascicle was separated and turned laterally and superiorly towards the motor branch of biceps and coated with 10-0 Ethilon without tension. 

During the second stage, the upper limb was moved over the patient’s chest and a posterior incision 10 cm long was made beginning at the scapula lateral border. The axillary nerve was dissected and sectioned proximal to the teres minor branch. Then the triceps lateral head motor branch of radial nerve was dissected, sectioned, and ﬂipped 180° to be coapted to the axillary nerve under the microscope using microsutures.

During the third stage, we changed patient position to lateral position, and a 12- to 15-cm long incision was made parallel to the spine of the scapula. Trapezius muscle was lifted up, and the spinal accessory nerve was isolated and taped. The upper border of the scapula was palpated for suprascapular notch and the suprascapular ligament was divided while protecting the underlying suprascapular nerve. The suprascapular nerve was mobilized proximally to allow sufficient length and coapted with distal spinal accessory nerve using 10-0 nylon suture. The standardized postoperative protocol was followed, including rehabilitation and regular physiotherapy when it was tolerated. 

Data collection and analysis 

Patients’ demography, intraoperative findings, and immediate postoperative outcome data were collected. Modified Narakas scale (Table 1) [11] was used to evaluate the shoulder function in addition to Waikakul scale (Table 2) [12] to evaluate the elbow function at 6, 12, 18, and 24 months for all tests. Data analysis produced median values; the range and mean of the functional scores where applicable. The data were recorded in an Excel spreadsheet and analyzed using SPSS statistical software (version 19; SPSS Inc, Chicago, IL, USA). Mann-Whitney U and Kruskal-Wallis tests of statistical hypotheses with p < 0.05 to indicate statistical significance were used throughout.

**Table 1:** Modified Narakas scale for shoulder function grading.

Grade	Functional status
Poor	No abduction movement and feeling of weightlessness in the limb [Medical Research Council (MRC) scale grade 0]
Fair	Stable shoulder without any subluxation but no active movement (MRC scale grade I)
Good	Active abduction of < 60 degrees (MRC scale grade III) and active external rotation of < 30 degrees
Excellent	Active abduction of > 60 degrees (MRC scale grade IV) and active external rotation of > 30 degrees

**Table 2:** Waikakul scale for elbow function grading.

Grade	Functional status
Excellent	Ability to lift 2 kg weight from 0 to 90 degrees of elbow flexion more than 30 times successively.
Good	Ability to lift 2 kg weight from 0 to 90 degrees of elbow flexion, but less than 30 repetitions successively.
Fair	Motor power more than MRC grade 3 power but unable to lift a 2 kg weight.
Poor	Motor power less than MRC grade 3.

## Results

Twenty-five patients (22 males and three females) with a median age at surgery of 29 years (20-50 years) were included in this study; all of them had triple neurotization and were followed up in our institute with a median follow up of two years. In the included patients, brachial plexus injury resulted from falling from a height, road traffic collision, and stab injuries. The right upper extremity was affected in 59% cases and left side in 41% cases. Intra-operative findings of the nerve roots status are summarized in Table 3. Minor postoperative complications included vomiting, and mild upper respiratory infection occurred in five cases (25%), and they all were successfully treated. 

**Table 3:** Summary of intraoperative findings.

Intraoperative finding	C5	C6	C7	C8	T1
Intact root	0	0	20	25	25
Avulsion	18	20	3	0	0
Conducting neuroma	2	0	0	0	0
Nonconducting neuroma	5	5	2	0	0
Total	25	25	25	25	25

Restoration of shoulder abduction assessed by modified Narakas scale [11] was excellent in 48% (n=12), good in 24% (n=6), fair in 16% (n=4), and poor in 12% of cases (n=3). Shoulder external rotation recovery assessed by modified Narakas scale [11] was excellent in 48% (n=12), good in 12% (n=3), fair in 12% (n=3), and poor in 28% (n=7) of cases. Recovery of elbow flexion assessed using Waikakul scale [12] was excellent in 60% (n=15), good in 12% (n=3), fair in 12% (n=3), and poor in 16% of cases (n=4). The mean postoperative value of shoulder abduction was 111.26 degrees (range: 70-150) while preoperatively none of the patients were able for shoulder abduction (p < 0.001). The mean postoperative value of shoulder external rotation was 57.5 degrees (range: 45-70) while preoperative range of shoulder external rotation was zero (p < 0.001). The mean postoperative value of elbow flexion was 75 degrees (range: 55-120) while preoperatively none of them were able for elbow flexion (p < 0.001).

## Discussion

Advanced reconstructive surgical options for brachial plexus injuries include nerve grafting, free-functioning muscle transfers, and tendon transfers. Attempts of single nerve transfer have been successful in restoring the function of the affected joint. To restore the shoulder function, the spinal accessory nerve was transferred to the suprascapular nerve with good results for shoulder abduction and acceptable results for external rotation function [13-16]. Songcharoen et al. [17] reported 80% motor recovery grade 3 according to the Medical Research Council (MRC) scale [18] with 60° of shoulder abduction and 45° of shoulder flexion in the transfer of 577 spinal accessory nerves to the suprascapular nerve. To enhance the restored shoulder function, dual nerve transfer of the spinal accessory nerve to suprascapular nerve and the triceps branch of the radial nerve to axillary nerve have been introduced [19]. Literature reports that results of restored shoulder external rotation function are inferior to shoulder abduction function [20-26]. To recover elbow flexion, intercostal nerves or the spinal accessory nerves were transferred to the biceps motor branch of the musculocutaneous nerve [17, 26]. Oberlin [27] described transferring ulnar nerve motor fascicles to biceps motor branch with 85% good results of MRC grade 3 or better biceps strength.

In this study, we observed acceptable functional outcomes for both elbow flexion and shoulder abduction and rotation among 25 patients following this technique. There are two other studies which specifically addressed this topic that of Bertelli and Ghizoni [28] and Leechavengvongs et al. [29]. Bertelli and Ghizoni [28] reported full elbow flexion recovery, 92 degrees average shoulder abduction recovery, and 93 degrees average shoulder external rotation restoration after using triple neurotization technique to reconstruct C5 C6 brachial plexus avulsion injury in 10 patients. Leechavengvongs et al. [29] used triple neurotization technique to reconstruct 15 cases of C5 C6 brachial plexus injury and reported full elbow flexion recovery, 115 degrees average shoulder abduction recovery, and shoulder external rotation recovery averaging 97 degrees. Our results are generally comparable with those two studies; although the elbow flexion and shoulder external rotation were lower in our series, our shoulder abduction restoration average was higher. 

We advocate a single procedure for triple surgery as not only it is very cost-effective, it also reduces exposure time to anesthesia and surgical stress with overall shorter hospital stay duration and rehabilitation time. This study, however, is not without its limitations. The sample size was relatively small, and not all of the data were collected prospectively; however, complete data collection was achieved in this cohort. This article only reports a single unit's practice and our results may not give a true reflection of outcomes that would be achieved in other units when using the same technique. We do, however, believe that our data support the safety and benefits of the application of triple neurotization as a single procedure for upper trunk brachial plexus injury, but more extensive studies from multiple centers are required.

## Conclusions

Brachial plexus injuries result in significant disabilities. Nerve transfers, including different donor nerves, have been introduced to reinnervate the paralyzed muscles and to restore the elbow and shoulder function. In our study, we transferred spinal accessory nerve to suprascapular nerve, ulnar fascicles to biceps branch of musculocutaneous nerve, and triceps long or lateral head branch to axillary nerve. The results of this study showed that the triple neurotization technique could be effective to restore elbow flexion and shoulder abduction and external rotation in adult patients with upper trunk brachial plexus injury. However, the main limitations of this study are the small sample size and included results from a single unit. Further multi-centred randomized controlled studies with bigger sample size are required to support this evidence.
